# Narrow microtunnel technology for the isolation and precise identification of axonal communication among distinct hippocampal subregion networks

**DOI:** 10.1371/journal.pone.0176868

**Published:** 2017-05-11

**Authors:** Udit Narula, Andres Ruiz, McKinley McQuaide, Thomas B. DeMarse, Bruce C. Wheeler, Gregory J. Brewer

**Affiliations:** 1 Biomedical Engineering, University of California Irvine, Irvine, California, United States of America; 2 J. Crayton Pruitt Family Department of Biomedical Engineering, University of Florida, Gainesville, Florida, United States of America; 3 Department of Pediatric Neurology, University of Florida, Gainesville, Florida, United States of America; 4 Department of Bioengineering, University of California San Diego, San Diego, California, United States of America; 5 MIND Institute, University of California Irvine, Irvine, California, United States of America; SUNY Downstate MC, UNITED STATES

## Abstract

Communication between different sub regions of the hippocampus is fundamental to learning and memory. However accurate knowledge about information transfer between sub regions from access to the activity in individual axons is lacking. MEMS devices with microtunnels connecting two sub networks have begun to approach this problem but the commonly used 10 μm wide tunnels frequently measure signals from multiple axons. To reduce this complexity, we compared polydimethylsiloxane (PDMS) microtunnel devices each with a separate tunnel width of 2.5, 5 or 10 μm bridging two wells aligned over a multi electrode array (MEA). Primary rat neurons were grown in the chambers with neurons from the dentate gyrus on one side and hippocampal CA3 on the other. After 2–3 weeks of culture, spontaneous activity in the axons inside the tunnels was recorded. We report electrophysiological, exploratory data analysis for feature clustering and visual evidence to support the expectation that 2.5 μm wide tunnels have fewer axons per tunnel and therefore more clearly delineated signals than 10 or 5 μm wide tunnels. Several measures indicated that fewer axons per electrode enabled more accurate detection of spikes. A clustering analysis comparing the variations of spike height and width for different tunnel widths revealed tighter clusters representing unique spikes with less height and width variation when measured in narrow tunnels. Wider tunnels tended toward more diffuse clusters from a continuum of spike heights and widths. Standard deviations for multiple cluster measures, such as Average Dissimilarity, Silhouette Value (S) and Separation Factor (average dissimilarity/S value), support a conclusion that 2.5 μm wide tunnels containing fewer axons enable more precise determination of individual action potential peaks, their propagation direction, timing, and information transfer between sub networks.

## 1. Introduction

The mammalian hippocampus plays an important role in the formation of long-term episodic memories and spatial navigation, yet encoding in sub-region for different elements of memory formation remains poorly understood. A better understanding of connectivity between different sub regions could aid novel computer designs, improve brain-computer interfaces and new approaches to restoring damaged brain circuits. Micro-tunnels that can approach single axon resolution and identification of connectivity between sub-regions would clarify information transmission between subregions.

Micro tunnel technology provides highly restrictive paths for connecting axons between different sub subnetworks. The first work in this area was done by [1] involving 3 chambers and a scratched collagen surface for guiding axon growth between primary cultures of sympathetic neurons. Similar designs to guide cortical and hippocampal axons required 10 μm wide tunnels [2] through a transparent PDMS design with two compartments connected to each other through these microfluidic tunnels [3]. The electrophysiological activity inside the tunnels can be measured by a multi electrode array (MEA), integrated below the microtunnel devices [4]. Tunnel widths have not been systematically varied to improve detection performance. As the width of a tunnel decreases, resistance increases, which causes an increase in the spike amplitude [5]. The tunnel-electrode construct allows simultaneous recordings with μm spatial and μs temporal resolution from a network of axons communicating between two sub regions. Thus, the flow of information between sub regions through the microtunnels could enable the acquisition of precise knowledge of the essential output from one region as the input to the next. Key features of the information transfer are spike direction of propagation from the timing difference on the two electrodes in the same tunnel, typically spaced 200 μm apart and spike and burst dynamics to decode the information transfer. However, these advantages are predicated on well-isolated spikes.

**Fig 1:**
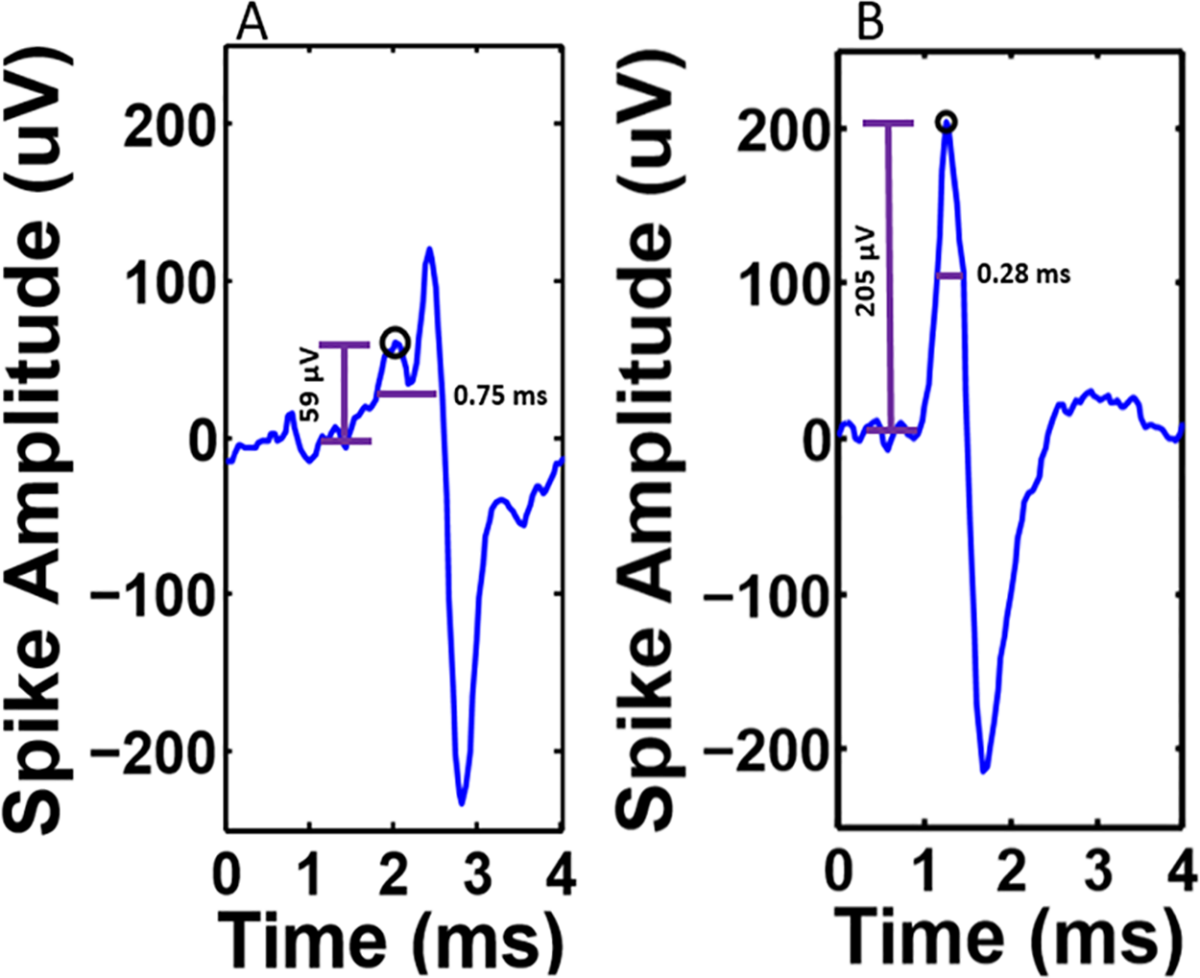
Complex spikes from multiple axons recorded on the same electrode are a problem for accurately determining spike height and peak time. (A) Example of distortion in a complex spike, more commonly observed in 10 μm tunnels (A) due to destructive interference from the contribution of two action potentials recorded by the same electrode. Note, a smaller but sufficient amplitude peak (59 μV, circle) that exceeds 9x the noise threshold with larger width at half-height (0.75 ms) due to the sum of action potentials from two spikes shifted about 0.5 ms. As a result, the larger amplitude signal failed detection because of the 1.6 ms dead time imposed in the spike detection algorithm. (B) The goal for accurate detection of width and timing of a well-isolated spike, here from the same electrode at a later time with larger height (205 μV) and smaller width (0.28 ms). We investigated whether narrower tunnels would favor increased detection of clean spikes and decrease complex spikes.

In open well recording in a medium with a spreading resistance from an electrode of 15 kOhm, somal currents overwhelm the low current source density of axons [4]. Inside tunnels, the increased resistance of 9.6 MOhm increases the axonal signal amplitude from 12 to 200 μV [4] and even to mV levels [6], which readily allow precise detection of axon signals from propagating action potentials. Without either a single axon per tunnel or infrequent spikes on two or more axons, complex spikes can lead to confusion. Fig 1A shows the features of a complex spike from two axons in the same tunnel with constructive and destructive interference. The result causes detection of a smaller spike amplitude and larger width, due to destructive interference of two action potentials within 0.5 ms peak detected on one electrode. The ideal case is shown in Fig 1B for the same larger action potential on the same electrode, but isolated from activity of the second axon at a later time with a higher peak amplitude and narrower peak width. The goal of this paper is to increase the percent of these well-isolated spikes and decrease the fraction of complex spikes. Spikes recorded from multiple axons pose sorting problems from this interference, but an approach favoring single axons enable clean distinction of the waveforms. Here, we propose to reduce the number of axons passing over the electrodes in tunnels to improve precision in the μs timing information of spikes passing between two sub networks. Relationships of network communication require knowledge of timing directions in inter-network communication, which is also achieved by axon isolation.

In this paper we report conclusions from an experiment asking whether narrower width of 5 or 2.5 μm, would maximize the detection of well-isolated spikes within more restricted clusters of height and width types. This would allow us to more accurately measure directionality from the timing of spikes from single axons passing over electrodes spaced 200 μm apart and improve the accuracy of the spike timing dynamics. To provide confidence of better axon isolation, a novel clustering approach was used to determine the relationship between tunnel width and the variation of spike height and width. In total, we used 27, 32 and 29 electrodes for 2.5, 5 and 10 μm tunnels (from 5 cultures). We report exploratory analysis of the electrophysiological data, using clustering of features extracted from action potential waveforms, augmented by visual confirmation, to show that narrower tunnels of 2.5 μm (in comparison to 5 or 10 um wide tunnels) allow passage of fewer axons per tunnel plus more accurate assignments of peak times and amplitudes.

## 2. Methods

### 2.1 Fabrication of microtunnel devices

**Fig 2:**
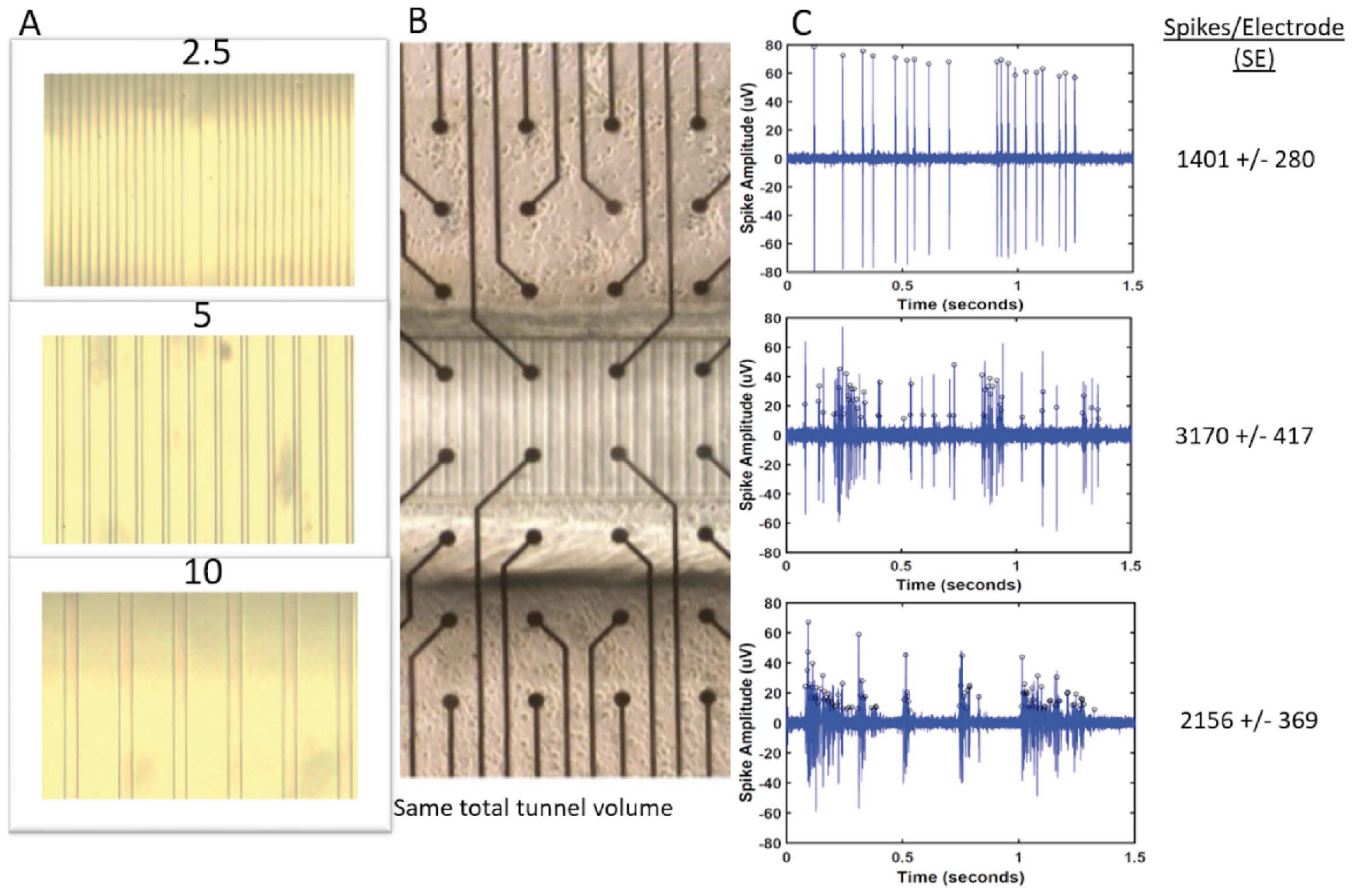
Microtunnel and MEA assembly with burst examples. (A) Tunnel widths of either 2.5, 5 or 10 μm were created in PDMS devices separating two chambers. The number of tunnels was adjusted for equal volume. (B) The devices were aligned over the 2 middle rows of an MEA (inter-electrode spacing 200 μm). (C) Bursts in different tunnel widths 2.5, 5 and 10 μm, with spike peaks represented by black circles. Mean and S.E. of spikes per electrode are shown. Note more uniform heights of higher amplitude in the 2.5 μm wide tunnels.

All tunnels were 800 μm long to preclude edge alignment problems, but truncated at 400 μm long by the separation of two compartments (Fig 2). A quartz microtunnel mask (Photo Science Inc., Torrance, CA) was designed with 2.5, 5 and 10 μm wide tunnels. To keep the tunnel volume constant, the 51 tunnels of 10 μm was doubled and quadrupled for the 5 and 2.5 μm wide tunnels. The devices were designed for the use with the MEA60 from MCS (Multi Channel Systems, Reutlingen, Germany), which have 30 μm diameter electrodes with 200 μm inter-electrode spacing. The mold fabrication process comprises the formation of two layers of SU-8: a thin 3 μm SU-8 structure of the microtunnels first and a second 0.5 mm thick SU-8 structure for forming the culture wells, improved from our earlier report [4]. We fabricated molds on a 4 inch single-side polished silicon (Si) wafer. The wafer was cleaned by piranha solution (H_2_SO_4_: H_2_O_2_ = 3:1) for 15 min, followed by rinsing in deionized water for 10 min. This was followed by submerging the wafer in buffered oxide etch (BOE) for 180 seconds and in deionized water for 10min, then heated on a hotplate at 125°C. Hexamethyldisilazane (HMDS) was applied to enhance adhesion of the photoresist. Positive photoresist S1813 (Microchem, Westborough, MA) was applied to the coated surface at a thickness of 1.3 μm by a Suss Delta 80 automatic spinner. The photoresist was soft-baked for 2 min at 112°C. A Karl Suss MA6 was used to expose the mask pattern in the S1813 with i-line(365 nm) exposure dosage of 143 mJ/cm^2^. In order to obtain high resolution patterning, vacuum exposure mode was used. To obtain the microtunnel structure, the photoresist was developed in 100 mL in a beaker with mild agitation for 75 sec, followed by a spray and wash with fresh developer for approximately 10 seconds, followed by a second rinse in deionized water for another 10 seconds. The microtunnel pattern was air dried with filtered, pressurized nitrogen. For superior tunnel definition, deep reactive ion etching (DRIE) was performed to obtain Si microtunnels with a depth of 2.5 μm. The remaining photoresist was removed with acetone.

After fabrication of the microtunnels, the microwells were fashioned. HMDS was re-applied and SU-8 2050 (Microchem) was poured on the substrate to a thickness of 0.5 mm, determined as SU-8 weight from the area times thickness time density divided by the solid concentration. Then the wafer was made planar at 65°C for 3 hr. with 2°C/min ramping, then ramped to 95°C for 24 hr. at 2°C/min. The nascent microwell pattern was aligned on a Karl Suss MA6 with the microwell film mask (CAD/Art Services Inc., Bandon, OR) and exposed for 5.2 min with 8 mW/cm2@365 nm in hard contact mode. The mold was post-exposure baked at 95°C for 1 hr. with 2°C/min ramping. The final pattern was obtained by immersing the substrate in 200 ml of SU-8 developer in a wide beaker with strong agitation for 40 min. followed by a spray and wash with fresh developer for approximately 10 seconds. The mold was cleaned with a spray and wash with isopropyl alcohol for 10 seconds, then rinses with deionized water for 10 seconds. Finally, the mold was air dry with filtered, pressurized air or nitrogen.

In order to facilitate release of PDMS devices from the molds, the mold was silanized in a desiccator in the presence of 20 μL 2-Methoxy(polyethyleneoxy propyl)trimethoxysilane (Gelest, San Diego, CA), 2–15 hr. Degassed PDMS along with curing agent (10:1 ratio; Dow Corning, Bay City, MI) was poured over the whole wafer surface inside an aluminum foil boat. After removing bubbles in a vacuum chamber, a 0.05 mm thick layer of PET plastic was pressed onto the PDMS over the wafer, weighted (approximately 50 gm, so as to rest on the tops of the chambers. The assembly was placed in a 60°C oven for 45 minutes. After cooling to room temperature, the weights were removed and plastic cover carefully peeled off. A 1 cm hole was punched to release each of 15 devices with tunnels as their center.

Each device was aligned on an MEA using a mask alignment microscope. The MEA is attached to a metal plate, via vacuum suction; the device is held by 10 μL ethanol acting as a release layer on a glass cover slip. In close proximity, the center two rows of the MEA were aligned over the tunnels. When a dark contact pattern was seen, indicating contact, the device released from the coverslip and adhered to the MEA, perfectly aligning the tunnels to the desired electrodes.

To promote adhesion of the neurons and passage of the axons through the tunnels, poly-D-Lysine in water (100 μg/ml; solution abbreviated as PDL), was applied, (P6407, Sigma-Aldrich, St. Louis, MO). First we put 6 μL ethanol on one side of the chamber, and then the other side to wet the tunnels and avoid trapping bubbles. Then ethanol from one chamber is aspirated and immediately replaced by PDL and followed by removal of ethanol from the other side to cause the PDL to be pulled through the tunnel by gravity. After 10 minutes, PDL was added to the other chamber. To make sure that ethanol is not present in the chambers, PDL in each chamber is replaced by fresh PDL. The devices were kept covered overnight at room temperature in the sterile hood. The next day, PDL was aspirated, replaced with water and aspirated again. The devices were then left to dry for 3 hours. Once dried, 6 μL NbActiv4 culture medium (BrainBits, Springfield, Illinois) + Gentamycin (100 μg/ml, (Gibco-ThermoFisher, NY) was pipetted into one chamber and then the other chamber and the device was placed in the incubator.

### 2.2 Cell culture

The use of rats after hypothermic or carbon dioxide asphyxiation was approved by the University of California Irvine IACUC and performed according to NIH guidelines on care and use of animals in research. Postnatal day 3 Sprague-Dawley rats (Charles Rivers Labs, San Diego) were anesthetized, brains removed and placed into 2 ml Hibernate A/Glx minus calcium (BrainBits) in a 35 mm D dish. The CA3, DG subregions of 3 brains were dissected and transferred to a 15 ml tube filled with Hibernate A–Ca, Glutamax, which is a dipeptide substitute for L-glutamine at 4°C (1 μg/ml, Gibco-ThermoFisher, NY). The tube with tissue along with a tube filled with papain (2 mg/ml, BrainBits) were warmed at 30°C for 10 minutes. The tissue was then transferred into the papain tube and further incubated for another 10 minutes. The tissue was transferred in 1 ml Hibernate A/B27/Glutamax (BrainBits, Springfield, Illinois) and triturated using a fire polished 9” pipet (BrainBits), until most of the tissue was homogenized. Non-dispersed pieces were allowed to settle for 3 minutes. The supernatant was transferred to a new tube, diluted with 2 ml Hibernate A/B27/Glutamax and centrifuged for 1 minute at 200 G. The supernatant was discarded and the pellet with about 20μl of residual supernatant was not disturbed. The tube was flicked to disperse the cells and diluted with 50μl or 100μl NbActiv4+ gentamycin for DG and CA3, respectively. 10 μl of the concentrated cells were mixed with 10 μl 0.4% trypan blue (Sigma) for counting in a hemocytometer. After the counting, the cells were diluted in order to plate 10,000 cells per well (6,000 cells/mm^2^). The devices were removed from the incubator and the medium was aspirated from the two chambers leaving a small amount to keep the tunnels filled. Source DG cells were plated by gently pipetting 7 μl with a repeat action to ensure wetting the tunnel entrance. The devices were then kept in the incubator for 10 minutes. After that, CA3 cells were plated in the other chamber, by pipetting gently two times near the tunnel entrance, and incubated for 30 minutes. Once the cells attached, 160 μl warm CO_2_-equilibrated NbActiv4 + gentamycin was added. The cells were kept at 37°C with 9% O_2_ and 5% CO_2_, balance N_2_ in a humidified incubator (Thermo-Forma #3432, Marietta, OH). Evaporation was limited by a Teflon sheet covering the MEA (ALA Scientific, Farmingdale, NY). 50% of the medium was changed twice a week with pre-equilibrated medium.

### 2.3 Recording and spike analysis

Recordings were made after 2–3 weeks in culture using Multi Electrode Arrays (MEA) from Multi Channel Systems (Reutlingen, Germany) with 60 TiN_3_ electrodes, one being ground, 30 μm in diameter and 200 μm apart. Signals were amplified at a gain of 1200x, sampled at 25 KHz, and kept at 37°C under sterile flow of 5% CO_2_, 9% O_2_, balance N_2_ (Airgas, Palmdale, CA). Spontaneous activity in the networks was recorded for 5 min. Data analysis was performed by using SpyCode v3.9 [7] along with custom MATLAB scripts (The MathWorks, Inc, Natick, Massachusetts). The central 2 rows of the MEA, with electrodes in the tunnels, were selected for analysis, filtered at 300 Hz high pass; spikes were identified as events whose peak to peak amplitudes exceeded 9 times the root mean square of a 200 ms contiguous window of samples. A refractory period of 1.6 ms was used. Threshold level was set for each electrode via the Graphical User Interface of SpyCode. SpyCode efficiently gives spike timing at the peak, but spike height and spike width were determined by custom MATLAB scripts. Out of all the spikes detected, only the positive arm of each spike was analyzed with an amplitude from 0 μV to the positive peak of spike. Spike widths were calculated at half height of the positive arm.

To analyze the effect of tunnel width on the variation in log10 values of spike height and width, a clustering approach was implemented. To determine the number of clusters present in each tunnel, three people inspected each spike height by width scatter plot (blinded to tunnel width). Based on the predominant cluster number, the MATLAB K-medoid function and custom scripts were used to characterize clusters. This analysis not only analyzed spike height and width variations in individual clusters, but also the separation of the clusters. Plots of height against width revealed tighter clusters with less variation in spike height and width, whereas diffuse clusters arose from higher variation. A quantitative measure of each cluster was determined as average dissimilarity (average of sum of all the distances from each point to the medoid in their respective clusters). Another measure was the Silhouette Value (S) of how similar an object is to its own cluster compared to other clusters). A third measure was Separation Factor (average dissimilarity divided by S value). The variation of each of the measure signifies the variation in spike height and width. Spike velocity was calculated by dividing the distance between two electrodes (200 μm) in a tunnel by the difference in spike times. Velocities from 0.2–0.83 m/s were considered as paired spikes; spikes under 0.2 m/s were considered ambiguous due to likely detection of spikes from two axons. Velocities above 0.83 m/s are termed as unpaired. [8]

### 2.4 Tunnel imaging

In order to image axons inside the microtunnels, the microtunnel devices were attached to 15 mm glass cover slips (German glass, 0.22 mm, Fisher Scientific), coated with PDL. The cells were grown in the two chambers in the same way as in the devices on MEAs. Imaging of axons inside the tunnels was facilitated by replacing the medium in the chamber containing the DG sub region with 2 μl Calcein AM (Thermo Fisher Scientific, Carlsbad, CA, diluted to 1 μg/500 μl NbActiv4). After an incubation of 20 min at 37°C in the CO_2_ incubator, the chamber with DG was rinsed with Hibernate A LF/glx at 37°C (BrainBits) and then the whole device with Hibernate A LF/glx. Axons were imaged by confocal microscopy (LSM 510 Zeiss, Munich, Germany) at 60x magnification with a 488 nm argon laser with a scan time of 4 seconds. The images were obtained with an optimum z-slice interval of 0.1 μm. A stack of 6 images was used to create an image containing small standard deviation of the intensities at each pixel, which eliminates noisy pixels. Analysis was done using ImageJ v1.63r [9].

## 3. Results

### 3.1 Spike height and width variation in terms of clusters

**Fig 3:**
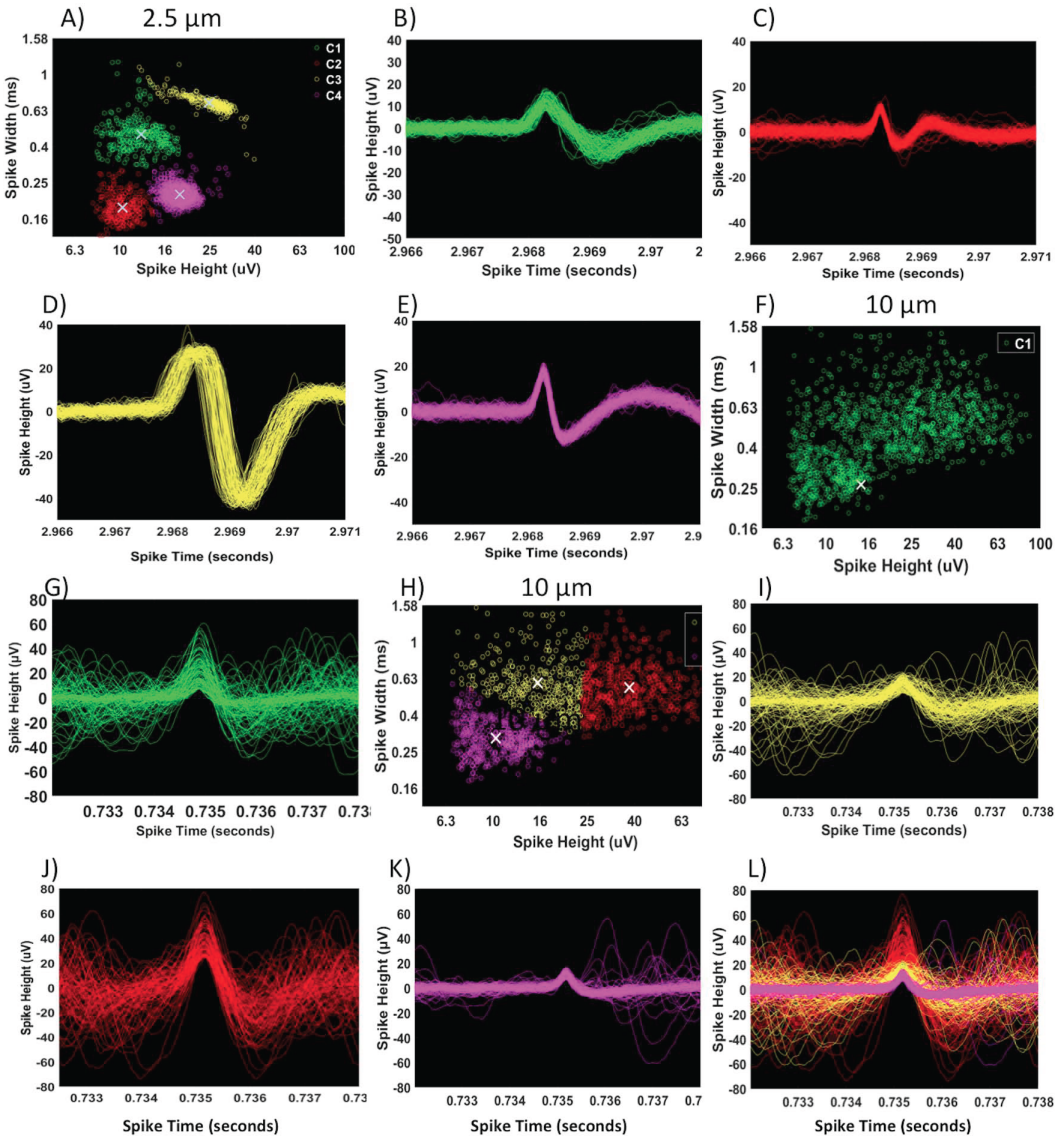
Tighter clusters of spike waveforms classified by spike height and width in narrow 2.5 μm compared to 10 μm wide tunnels. (A) 2.5 μm wide tunnel with four tight clusters, putatively from four different axons in one 2.5 μm wide tunnel. X marks the medoid of each cluster. Total spikes in each cluster: C1- 313, C2- 271, C3- 278, C4- 1138. (B-E) Tight alignment of the waveforms of each cluster of the first 100 spikes shown for clarity. (F) 10 μm tunnel of a diffuse cluster of spike waveforms difficult to separetely classify. (G) First 100 spikes out of 2212 are shown, which are aligned poorly. (H) Forced clustering of data from (F) failed to show discrete waveforms. (I, J and K) show poor classification in first 100 waveforms of each “cluster”. (L) Note that the composite of I, J and K produced a continuum of waveform heights and widths.

We used the same tunnel volume for each of the 2.5, 5 and 10 μm tunnel designs to control for a constant amount of communication between the two compartments. To determine whether narrow tunnels produced a higher yield of isolated action potentials from fewer axons, we used the K-medoid function of MATLAB to sort and classify spike width and height characteristics. We hypothesized that a single axon in one tunnel would produce spikes of similar heights and widths which would tightly cluster feature space and that other axons in the same tunnel would also have tight but distinguishable clusters. Conversely, wider tunnels would have more axons and more overlapping action potentials leading to more diffuse clusters. To validate the clustering algorithm based on spike height and width features, we examined the waveforms for each cluster. Fig 3A shows a cluster plot for a 2.5 μm tunnel with a worst case of four clusters. Waveforms for each cluster (B-E), aligned well over each other (first 100 spikes shown). The near perfect overlap of waveforms suggests that tightness of a cluster can be linked with consistency in spike height and width. Fig 3F shows a cluster plot for a 10 μm wide tunnel with one diffuse cluster. Waveforms for the diffuse cluster (G) are poorly aligned forming a continuum over a large range of spike heights and widths. If the clustering algorithm is forced to sort the feature data into 3 clusters (H), the wide continuum of heights and widths is more readily evident (I-K) in especially when recombined (L). With this better understanding of extreme examples of tight and diffuse clustering, we could next determine whether they were statistically associated with narrow and wide tunnels.

### 3.2 Number of clusters per tunnel and their properties

**Fig 4:**
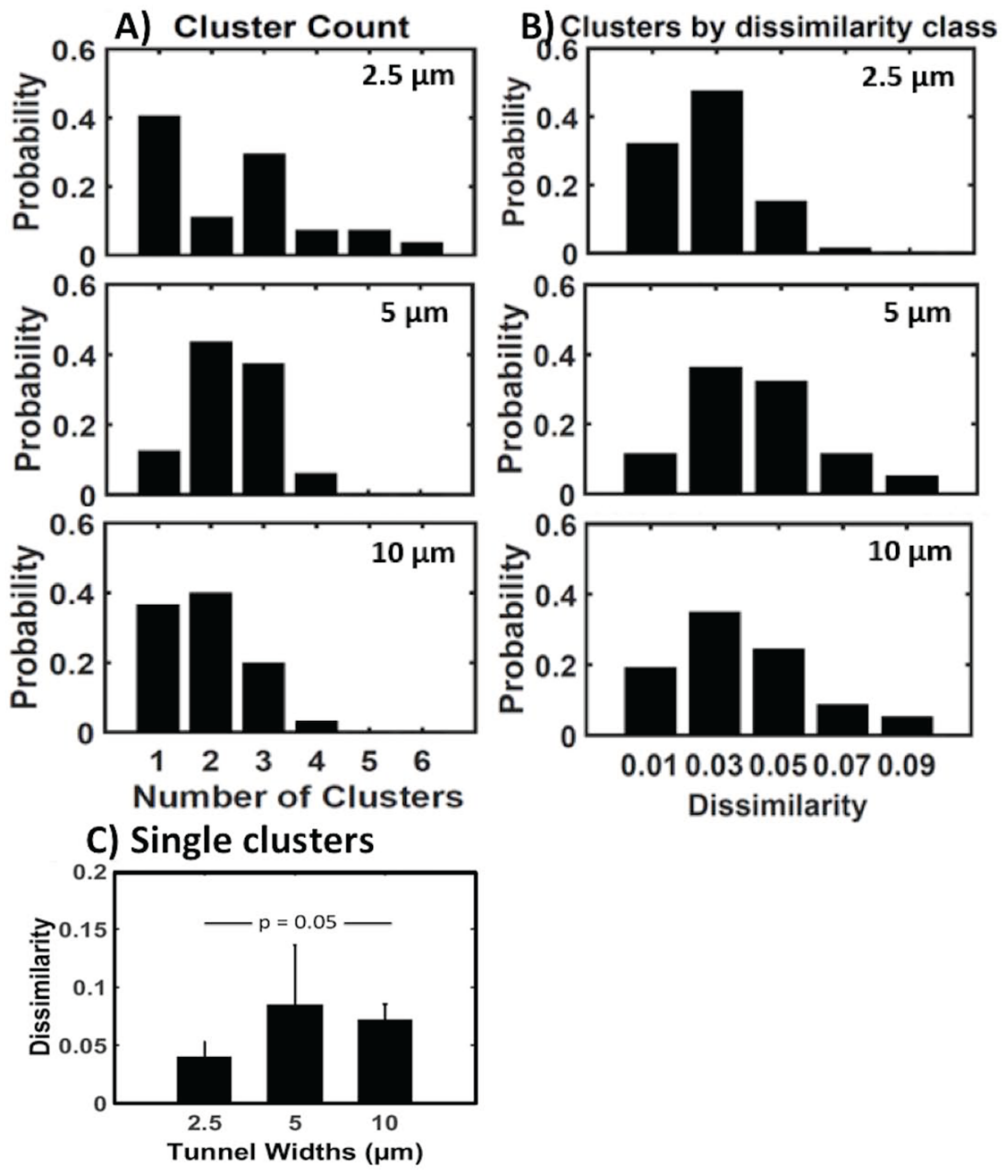
Variations in classification by spike height and width by tunnel width show advantage for narrower 2.5 μm tunnels. (A) Distribution of cluster count per tunnel for 2.5, 5 and 10 μm tunnels. (B) Distribution of the dissimilarity measure of cluster tightness or diffuseness shows shift to lower dissimilarity (tighter clusters) for 2.5 μm tunnels. (C) Dissimilarity measure for single clusters in 2.5, 5 and 10 μm tunnels show significantly better clustering for 2.5 μm tunnels by Wilcoxon signed-rank test.

We tested whether narrow tunnels would exhibit fewer clusters from fewer axons per tunnel than the wide tunnels. Fig 4A shows that one cluster per 2.5 μm wide tunnel was most prevalent, although by no means exclusive (mean 2.4 ± 0.3 clusters per tunnel). For 5 μm wide tunnels, 2 or 3 clusters were most prevalent (mean 2.4 ± 0.1). Surprisingly, single clusters were nearly as prevalent in 10 μm wide tunnels as in 2.5 μm wide tunnels, although 2 clusters were also common (mean 1.9 ± 0.2). The mean cluster count was not significantly different (F(2,86) = 2.13, p = 0.125). From Fig 3F, we considered whether a single diffuse cluster could arise from constructive and destructive interference of multiple action potentials from multiple axons, which would compromise the precision with which we could estimate spike timing (Fig 3F–3L).

To observe whether tunnel width affected the tightness or diffuseness of the distributions of action potentials widths and heights, we determined the distribution of a dissimilarity measure of the clusters from each tunnel. Dissimilarity is the sum of all the distances from each point to the medoid in each cluster divided by the number of points (spikes). Fig 4B shows this distribution of dissimilarities per tunnel with 2.5 μm tunnels having 50% higher number of tight clusters than wider tunnels (5 and 10 μm). Conversely, 5 and 10 μm wide tunnels produced more diffuse clusters. Since Fig 4A shows a prominent class of 41% single clusters for 2.5 μm wide tunnels among all clusters in Fig 4B, we calculated the average dissimilarity for single clusters (Fig 4C). In Fig 4C, the average dissimilarity of single clusters in 10 μm wide tunnels was 0.07 ±0.01, indicating a diffuse type, compared to 0.04 ±0.01 in 2.5 μm wide tunnels, indicating a tight cluster type (Wilcoxon signed-rank test, p = 0.05). These results suggest that 2.5 μm tunnels produce more single tight clusters than those produced by wider tunnels and the single clusters seen in 10 μm wide tunnels were more often diffuse.

### 3.3 Better segregation of tight clusters in 2.5 μm tunnels from diffuse clusters in 5 and 10 μm wide tunnels

**Fig 5:**
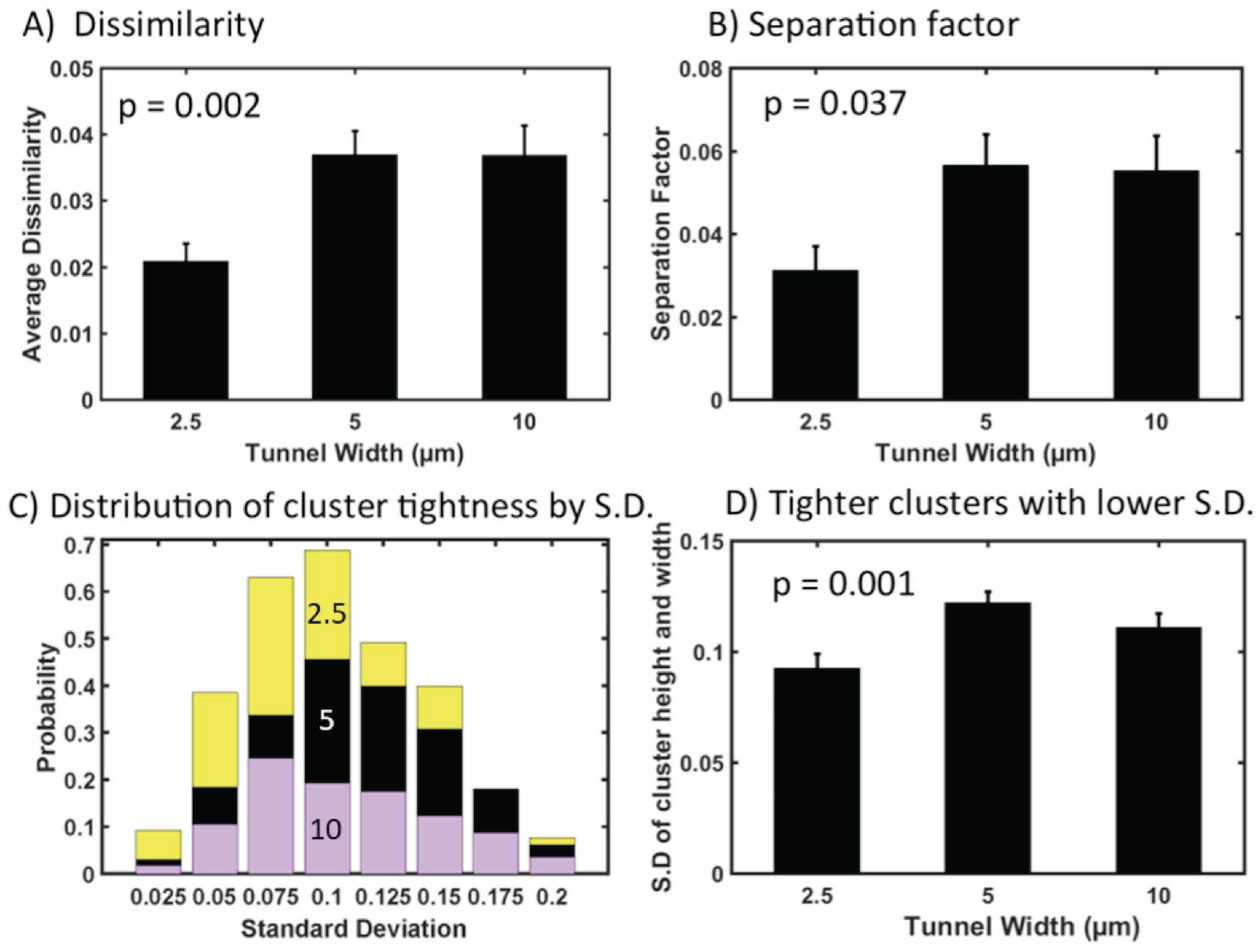
Four measures of cluster tightness indicate best performance for 2.5 μm tunnels. (A) Lower average dissimilarity between the observations in the cluster and the cluster’s medoid for 2.5 μm tunnels. (B) Separation Factor as the ratio of average dissimilarity to silhouette value favors 2.5 μm tunnels. (C) Distribution of the clusters at each standard deviation for different tunnel widths. Note lower standard deviations for 2.5 μm tunnels. (D) Standard deviation of spike widths and heights for each cluster by tunnel width favors lower deviations for 2.5 μm tunnels.

From the distributions of dissimilarities in Fig 4B, we determined in Fig 5A the average dissimilarity of each cluster and their dispersion as standard error. Average dissimilarity is the sum of all the distances from each point to the medoid in their respective clusters divided by the number of points (spikes). It represents how tight or diffuse a cluster is for individual clusters only. Fig 5A shows that spike clusters were significantly tighter (more consistency in spike height and width) for 2.5 μm tunnels than wider tunnels (F(2,196) = 6.2, p = 0.002).

To evaluate the separation between clusters, the silhouette metric determines how similar an object is to objects in other clusters; the average silhouette value over a group of clusters is a measure of the segregation of clusters from other clusters. Silhouette values for data from multiple clusters were similar for the three tunnel classes: 0.74 ± 0.03, 0.71 ± 0.03, 0.72 ± 0.02, for 2.5, 5 and 10 μm tunnels, respectively (p = 0.66). Although 2.5 μm tunnels produced higher silhouette values than the others, the failure to find significant separation probably resulted from the many cases where only a single cluster appeared in the data (an undefined state).

To better adjust for cases with just one cluster, a new metric of separation factor was derived. This “Separation factor” was defined as the ratio of average dissimilarity and silhouette value of respective clusters. The silhouette value is set at 1 for data with only one cluster. Evaluation of separation factor (Fig 5B) shows a significant decrease in value for 2.5 μm tunnels compared to wider tunnels (F(2,86) = 3.4, p = 0.037), suggesting better separation of axon spike times. The distribution of the clusters of spike types (Fig 5C) showed a shift to tighter (lower) deviations from the medoid for 2.5 μm tunnels (yellow), compared to 5 and 10 μm tunnels. Compared to wider tunnels, Fig 5D shows that 2.5 μm tunnel data exhibit a lower standard deviation (F(2,195) = 6.79,p = 0.001), further supporting the hypothesis that clusters are tighter in 2.5 μm wide tunnels.

### 3.4 No difference in conduction velocities with tunnel width

Because we have 2 electrodes in each tunnel, we can also estimate the speed and direction of propagation of any action potentials propagating through the tunnels. Directional propagation from DG to CA3 was 64, 67 and 64% of paired spikes from 2.5, 5 and 10 μm tunnels, respectively. This directionality is in agreement with previous results in 10 μm wide tunnels [8]. From 2.5, 5 and 10 μm tunnels, we detected insignificantly different average speeds of 0.43 ± 0.04, 0.48 ± 0.05 and 0.38 ± 0.02 m/s respectively in the predominant direction (DG->CA3) (F(2,46) = 2.05, p = 0.14). The speed in the opposite direction was 0.63 ± 0.06, 0.48 ± 0.03, 0.47 ± 0.06. These results suggest that the axon diameter is similar in each tunnel width. The observed speeds are consistent with the extra-burst measures of 0.51 m/s reported previously [10].

### 3.5 Lower axon count in narrow tunnels by confocal microscopy

**Fig 6:**
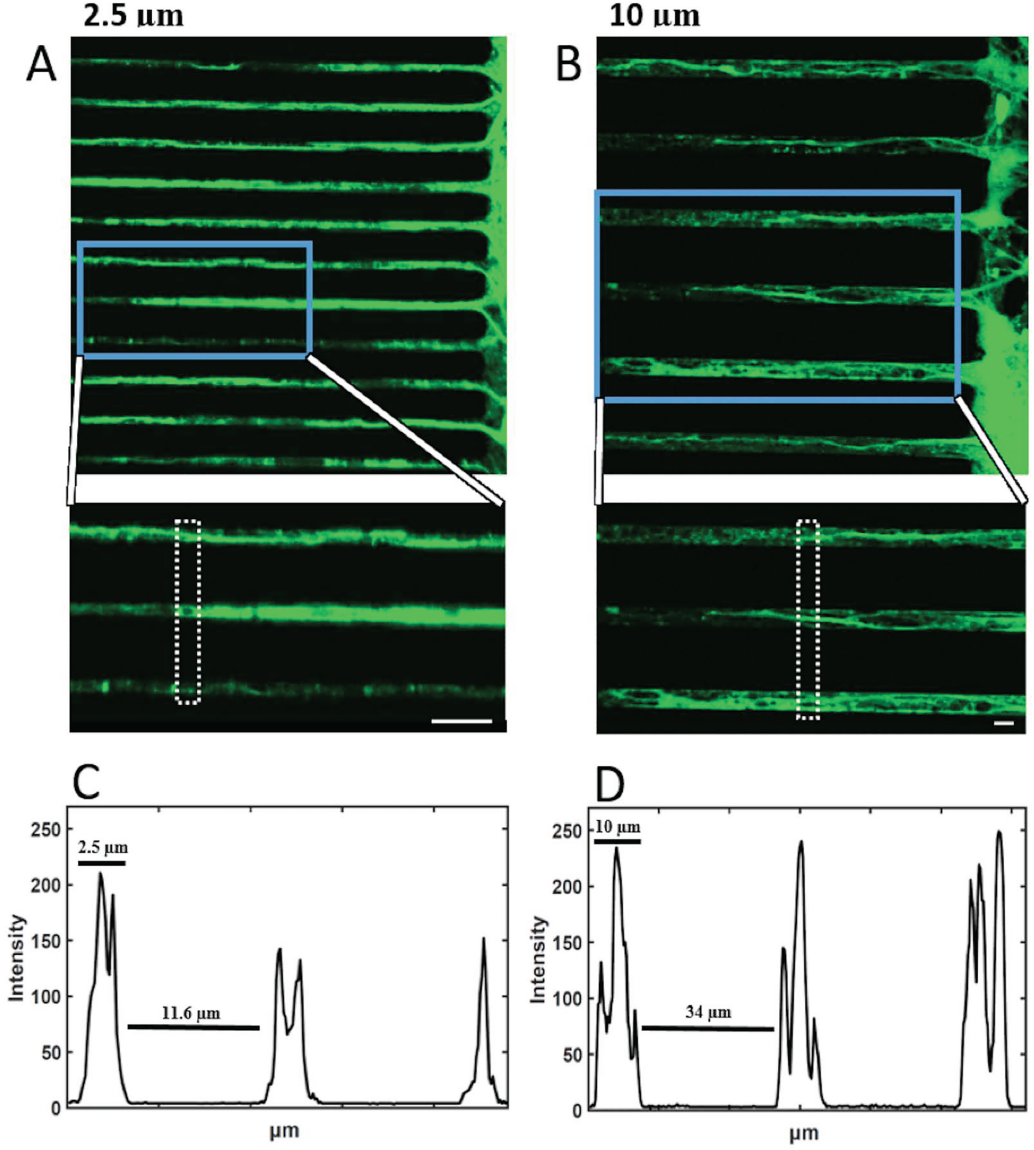
Confocal imaging shows lower axon count in narrow tunnels. In comparison with (A) 2.5 μm tunnels, (B) 10 μm tunnels show higher axon count (average of 3 axons per tunnel). Scale bar = 10 μm (A and B). (C) Intensity plots along the dotted sections of the enlarged area in (A) (2.5 μm) show 2, 2 and 1 axons. (D) Similar profile for enlarged area in B (10 μm) shows clear evidence for 3, 3 and 3 axons.

Confocal images were taken from 3 week cultures for visual evidence of fewer axons in narrow tunnels. As seen in the image in Fig 6A and 6C one or two axons were seen inside 2.5 μm tunnels, whereas in 10 μm tunnels (B), two to three or more axons are readily resolved (6B, D). This suggests that 2.5 μm width is sufficient to greatly reduce the number of axons but not to reliably constrain these narrow tunnels to a single axon.

## 4. Discussion

Multiple neurons or axons per electrode leads to variations in recorded spike amplitudes and widths contributing to imprecise estimates of spike timing. We attempted to solve this problem by isolation of single axons for greater spike timing precision. For the first time, we created microtunnels with narrow widths of 2.5 and 5 μm to systematically compare with the more common 10 μm wide tunnels. Compared to 10 μm wide tunnels, spikes recorded from 2.5 μm wide tunnels exhibited spike heights and widths that clustered into groups with smaller average dissimilarity and greater separation; the spike waveforms from the smaller tunnels could also be better aligned. Confocal imaging gave visual evidence that fewer axons grew in the narrow 2.5 μm tunnels than in the 10 μm wide tunnels, again supporting our hypothesis. However, we were unable to consistently isolate single axons. Hippocampal axon width is approximately 0.8 μm [11], which means that even the narrow tunnel width of 2.5 μm could accommodate up to 9 axons with three in direct contact with the 30 μm electrode. In one case of 2.5 μm tunnel we observed a maximum of 6 tight clusters, suggesting 6 axons. In the future, we may be able to isolate single axons by reducing the plating density of cells at the expense of unoccupied tunnels and lower network activity. Another approach to isolate single axons would be to decrease the tunnel width to 1 μm, which is achievable with UV-laser light [12].

Our understanding of learning and memory could be greatly improved if we were able to achieve access to communication among neuronal networks of different sub regions of neurons with simultaneous access to inter-network/inter-regional communication [13]. Axon-selective microtunnels of 10 μm width were constructed to visualize bundles of axons [3, 14] and for analysis of CNS axonal injury and regeneration [15]. Other work with two different brain regions of thalamus and cortex followed later [16], but did not monitor axon activity with electrodes in the tunnels as in our work with pairs of DG-CA3 and CA3-CA1 [8]. Electrical activity of the axons was recorded from a single electrode [17] and multisites [4, 8, 18] through the tunnels with higher signal-to-noise ratio value [5] than conventional open-access substrate-embedded microelectrodes. Horse serum was also used to promote astroglial growth, which likely causes clogging of the tunnel entrance and increases the voltage recordings as high as 1 mV [4, 17]. As Claverol-Tinture et al. [18] decreased the cross sectional dimensions of tunnels from 3.5 x 25 μm (92 μm^2^) to 1 x 5 μm (5 μm^2^), the variability in spike height increased but the median height did not significantly increase. We suspect that they failed to demonstrate increased height because they did not examine clusters based on spike height and width, as we did here. Also, they reported no significant difference in the signal-to-noise for the different dimension tunnels, which is consistent with our results (13, 11 and 14 for 2.5, 5 and 10 μm tunnels respectively). Spike propagation direction and speed were further examined [6, 8] to better understand the connectivity between different sub regions of the hippocampus in 10 μm wide and now 2.5 μm wide tunnels. Since the structural connectivity via the number of microtunnels between the two networks (CA3 and DG) affects the formation and functional activity between them [19–21], we kept the volume constant for each of the widths to allow equal connectivity with minimal effects on the network sending axons into the tunnels. None of this previous work has led to isolation of single axons in microtunnels, which we have approached in this paper using narrow tunnels.

We also consider whether multiple axons in a narrow tunnel would promote ephaptic coupling [22]. In the case where unmyelinated axons are present, as in microtunnels [6], one could hypothesize that the membranes of pairs of axons could be tightly coupled even near gap junction tightness. In such close proximity, an action potential in one axon could induce an action potential in another axon, by ephaptic coupling from highly localized electric fields or even ionically through local perturbation of sodium and potassium concentrations and resultant changes in equilibrium potentials. Thus, an action potential in one axon begins with sodium influx, quickly followed by potassium efflux. This first lowers the extracellular sodium and then raises potassium, both of which partially depolarize a nearby axon. The current from one impulse induces a longitudinal voltage in the external resistance, influencing the dynamics of an adjacent impulse [22]. Endogenous electric field activity produces only small 0.5 mV changes in adjacent axon potential, insufficient to depolarize. However, together with normal membrane fluctuations, this periodic 0.5 mV change is sufficient to entrain the timing of action potentials of adjacent axons [23]. The induced second action potential is delayed by 0.3–0.5 ms [24, 25], which is within our measured 0.2–0.8 ms range of spike widths. Hence, an ephaptic spike could cause spike overlap and broadening. This ephaptic coupling would be more likely to occur in narrow 2.5 μm tunnels, where two or three axons are in closer proximity than the same number in a 10 μm tunnel. Ephaptic coupling in narrow tunnels could induce a faster propagation speed than the regular spike because of higher field amplitude [25], which might explain a larger variation in the observed velocities. Overall, ephaptic coupling may be more prevalent in the narrow tunnels, but would need to be studied by single axon stimulation.

In conclusion, from analysis of spike waveforms, wider tunnels have more axons whose action potentials are more likely to interfere with the precise detection of spike timing compared to fewer axons in narrow tunnels. We have been able improve isolation of fewer axons inside the 2.5 μm tunnels compared to the wider microtunnels (10 μm). Our result is supported by electrophysiological tests, exploratory data analysis for feature clustering and visual evidence of the axons inside the microtunnels. Our work will help to decode the information flow between reconstituted brain sub regions, along with drug trials for neurological diseases with higher precision.

## References

[1] Campenot RB. Local control of neurite development by nerve growth factor. Proc Natl Acad Sci U S A. 1977;74:4516–9. 270699 10.1073/pnas.74.10.4516 PMC431975

[2] Taylor AM, Blurton-Jones M, Rhee SW, Cribbs DH, Cotman CW, Jeon NL. A microfluidic culture platform for CNS axonal injury, regeneration and transport. Nat Methods. 2005;2:599–605. 10.1038/nmeth777 16094385 PMC1558906

[3] Rhee SW, Taylor AM, Tu CH, Cribbs DH, Cotman CW, Jeon NL. Patterned cell culture inside microfluidic devices. Lab on a chip. 2005;5:102–7. 10.1039/b403091e 15616747

[4] Dworak BJ, Wheeler BC. Novel MEA platform with PDMS microtunnels enables the detection of action potential propagation from isolated axons in culture. Lab on a chip. 2009;9:404–10. 10.1039/b806689b 19156289 PMC2790813

[5] Wang L, Riss M, Buitrago JO, Claverol-Tinture E. Biophysics of microchannel-enabled neuron-electrode interfaces. J neural eng. 2012;9:026010 10.1088/1741-2560/9/2/026010 22333069

[6] Pan L, Alagapan S, Franca E, Brewer GJ, Wheeler BC. Propagation of action potential activity in a predefined microtunnel neural network. J neural eng. 2011;8:046031 10.1088/1741-2560/8/4/046031 21750372 PMC3213028

[7] Bologna LL, Pasquale V, Garofalo M, Gandolfo M, Baljon PL, Maccione A, et al Investigating neuronal activity by SPYCODE multi-channel data analyzer. Neural Networks. 2010;23:685–97. 10.1016/j.neunet.2010.05.002 20554151

[8] Brewer GJ, Boehler MD, Leondopulos S, Pan L, Alagapan S, DeMarse TB, et al Toward a self-wired active reconstruction of the hippocampal trisynaptic loop: DG-CA3. Frontiers in neural circuits. 2013;7:165 10.3389/fncir.2013.00165 24155693 PMC3800815

[9] Schneider CA, Rasband WS, Eliceiri KW. NIH Image to ImageJ: 25 years of image analysis. Nature methods. 2012;9:671–5. 22930834 10.1038/nmeth.2089 PMC5554542

[10] Bhattacharya A, Desai H, DeMarse TB, Wheeler BC, Brewer GJ. Repeating Spatial-Temporal Motifs of CA3 Activity Dependent on Engineered Inputs from Dentate Gyrus Neurons in Live Hippocampal Networks. Frontiers in neural circuits. 2016;10:45 10.3389/fncir.2016.00045 27445701 PMC4923256

[11] Bartlett WP, Banker GA. An electron microscopic study of the development of axons and dendrites by hippocampal neurons in culture. I. Cells which develop without intercellular contacts. The Journal of neuroscience: the official journal of the Society for Neuroscience. 1984;4:1944–53. 6470762 10.1523/JNEUROSCI.04-08-01944.1984 PMC6564955

[12] Ghosh S, Ananthasuresh GK. Single-photon-multi-layer-interference lithography for high-aspect-ratio and three-dimensional SU-8 micro-/nanostructures. Scientific Reports. 2016;6:18428 10.1038/srep18428 26725843 PMC4698723

[13] Berdondini L, Chiappalone M, van der Wal PD, Imfeld K, de Rooij NF, Koudelka-Hep M, et al A microelectrode array (MEA) integrated with clustering structures for investigating in vitro neurodynamics in confined interconnected sub-populations of neurons. Sensors and Actuators B: Chemical. 2006;114:530–41.

[14] Taylor AM, Rhee SW, Tu CH, Cribbs DH, Cotman CW, Jeon NL. Microfluidic multicompartment device for neuroscience research. Langmuir. 2003;19:1551–6. 10.1021/la026417v 20725530 PMC2923462

[15] Taylor AM, Blurton-Jones M, Rhee SW, Cribbs DH, Cotman CW, Jeon NL. A microfluidic culture platform for CNS axonal injury, regeneration and transport. Nature methods. 2005;2:599–605. 10.1038/nmeth777 16094385 PMC1558906

[16] Kanagasabapathi TT, Franco M, Barone RA, Martinoia S, Wadman WJ, Decre MM. Selective pharmacological manipulation of cortical-thalamic co-cultures in a dual-compartment device. Journal of neuroscience methods. 2013;214(1):1–8. 10.1016/j.jneumeth.2012.12.019 23305774

[17] Claverol-Tinture E, Ghirardi M, Fiumara F, Rosell X, Cabestany J. Multielectrode arrays with elastomeric microstructured overlays for extracellular recordings from patterned neurons. J neural eng. 2005;2:L1–7. 10.1088/1741-2560/2/2/L01 15928406

[18] Claverol-Tinture E, Cabestany J, Rosell X. Multisite recording of extracellular potentials produced by microchannel-confined neurons in-vitro. IEEE transactions on bio-medical engineering. 2007;54:331–5. 10.1109/TBME.2006.880903 17278590

[19] Volman V, Baruchi I, Ben-Jacob E. Manifestation of function-follow-form in cultured neuronal networks. Phys Biol. 2005;2:98–110. 10.1088/1478-3975/2/2/003 16204862

[20] Feinerman O, Segal M, Moses E. Identification and dynamics of spontaneous burst initiation zones in unidimensional neuronal cultures. JNeurophysiol. 2007;97:2937–2948. 17287439 10.1152/jn.00958.2006

[21] Pan L, Alagapan S, Franca E, Leondopulos SS, DeMarse TB, Brewer GJ, Wheeler BC. An in vitro method to manipulate the direction and functional strength between neural populations. Front Neural Circuits. 2015;9:32 10.3389/fncir.2015.00032 26236198 PMC4500931

[22] Binczak S, Eilbeck JC, Scott AC. Ephaptic coupling of myelinated nerve fibers. Physica D: Nonlinear Phenomena. 2001;148:159–74.

[23] Anastassiou CA, Perin R, Markram H, Koch C. Ephaptic coupling of cortical neurons. Nat Neurosci. 2011;14:217–23. 10.1038/nn.2727 21240273

[24] Katz B, Schmitt OH. Electric interaction between two adjacent nerve fibres. J Physiology. 1940;97:471–88. 10.1113/jphysiol.1940.sp003823 PMC1393925 16995178

[25] Qiu C, Shivacharan RS, Zhang M, Durand DM. Can Neural Activity Propagate by Endogenous Electrical Field? J Neuroscience. 2015;35:15800–11. 10.1523/JNEUROSCI.1045-15.2015 26631463 PMC4666910

